# Supplementary material for: Physical state of water controls friction of gabbro-built faults
Source: Nat Commun. 2023 Aug 8;14:4612. doi: 10.1038/s41467-023-40313-x (PMC10409772; doi:10.1038/s41467-023-40313-x)
Supplement: Supplementary file 1 — Supplementary Information [file 41467_2023_40313_MOESM1_ESM.pdf]

Supplementary information for "physical state of water controls friction of gabbro-built faults"

Wei Feng[1,2], Lu Yao[2]*, Chiara Cornelio[3], Rodrigo Gomila[1], Shengli Ma[2], Chaoqun Yang[4], Luigi Germinario[1], Claudio Mazzoli[1] and Giulio Di Toro[1,3]*

[1] Dipartimento di Geoscienze, Università degli Studi di Padova, Padua, Italy

[2]State Key Laboratory of Earthquake Dynamics, Institute of Geology, China Earthquake Administration, Beijing, China

[3]Sezione Roma 1, Istituto Nazionale di Geofisica e Vulcanologia, Rome, Italy

[4]State Key Laboratory of Oil and Gas Reservoir Geology and Exploitation, Chengdu University of Technology, Chengdu, China

*Corresponding authors: Lu Yao (yaolu_cug@163.com) and Giulio Di Toro (giulio.ditoro@unipd.it)

This file contains additional information about experimental configuration, experimental results, micro-analytical results, composition of gabbro, and numerical modeling results of thermal pressurization.

Contents of the file:

1. Supplementary Figures 1 to 10

2. Supplementary Tables 1 to 3

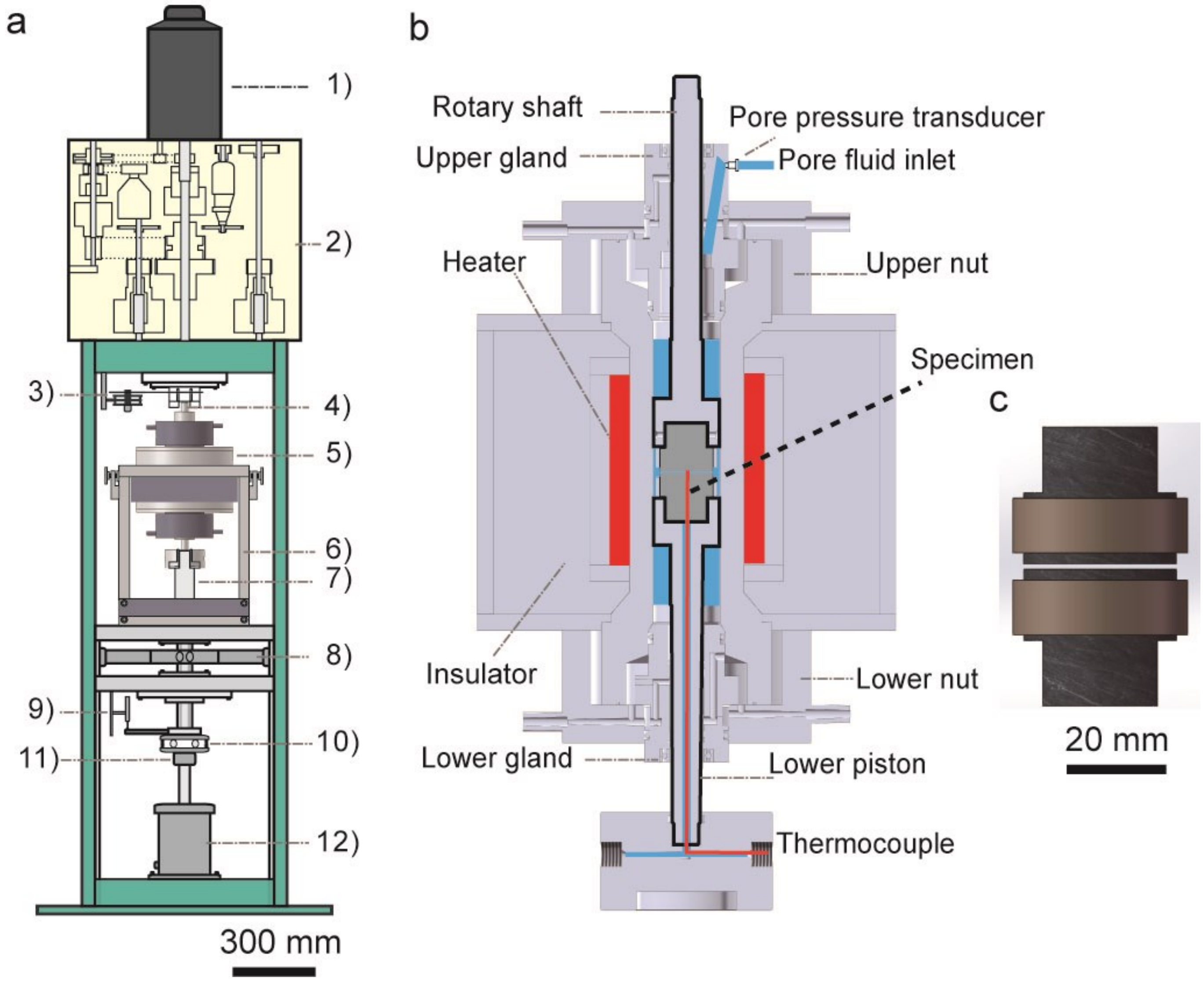

Supplementary Figure 1. Experimental setup. a. The Low to High velocity rotary shear apparatus, equipped with a dedicated hydrothermal vessel (modified from Ma et al., 2014[1]). 1) servomotor, 2) gear and belt system, 3) rotary encoder and potentiometer, 4) rotary shaft, 5) hydrothermal pressure vessel, 6) metal frame, 7) axial loading column, 8) cantilever-type torque gauge, 9) liner variable differential transducer, 10) thrust bearing, 11) axial force gauge, 12) air actuator. **b.** The sketch of the hydrothermal vessel with the main parts indicated in the diagram. **c.** Sample assembly: pair of gabbro cylinders jacketed by nickel rings for friction experiments.

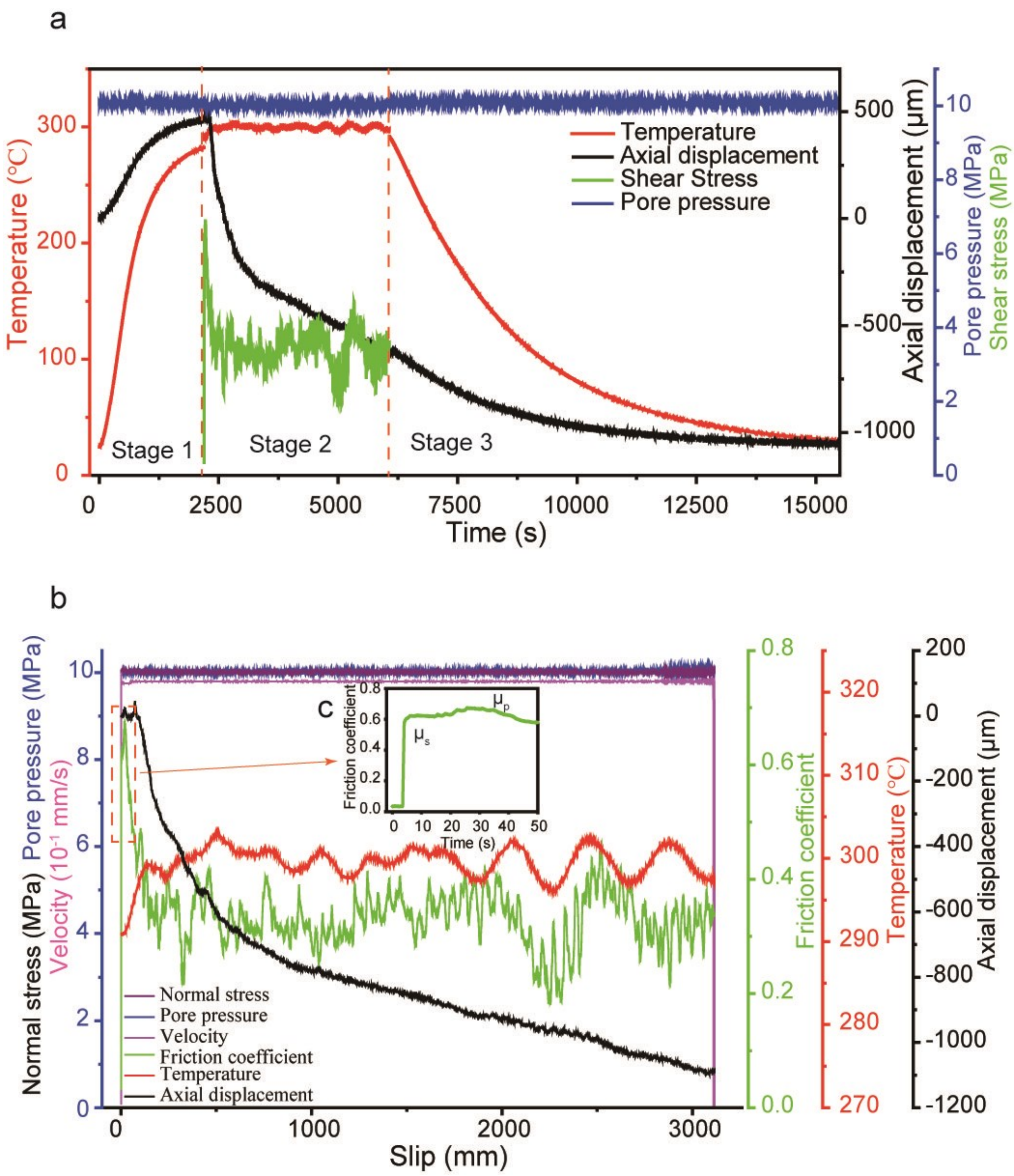

Supplementary Figure 2. **Representative experiment (LHV2359) conducted at effective normal stress σ_n^{eff} = 10 MPa, temperature T = 300°C, pore pressure P_f = 10MPa, slip velocity V =1 mm/s (i.e., water in the liquid state) showing the experimental procedure and mechanical data. a.** Temperature, axial displacement, shear stress and pore pressure versus time for the entire experiment. Stage 1: heating, the temperature increases to the target value. Stage 2: friction test, temperature and pore pressure are kept constant within 2% oscillation. Stage 3: quenching, the temperature cools down to room condition. **b.** Zoom of Stage 2 showing the normal stress, pore pressure, slip velocity, friction coefficient, temperature and axial displacement as a function of slip displacement. During slip, friction coefficient overcomes the static friction (μ_s) and increases to a peak value (μ_p), followed by a decay to the steady-state value (μ_{ss}). Axial displacement shortens faster in the running-in stage and evolves into steady state shortening. **c.** Zoom of the initial part of the slip with the definition of μ_s and μ_p.

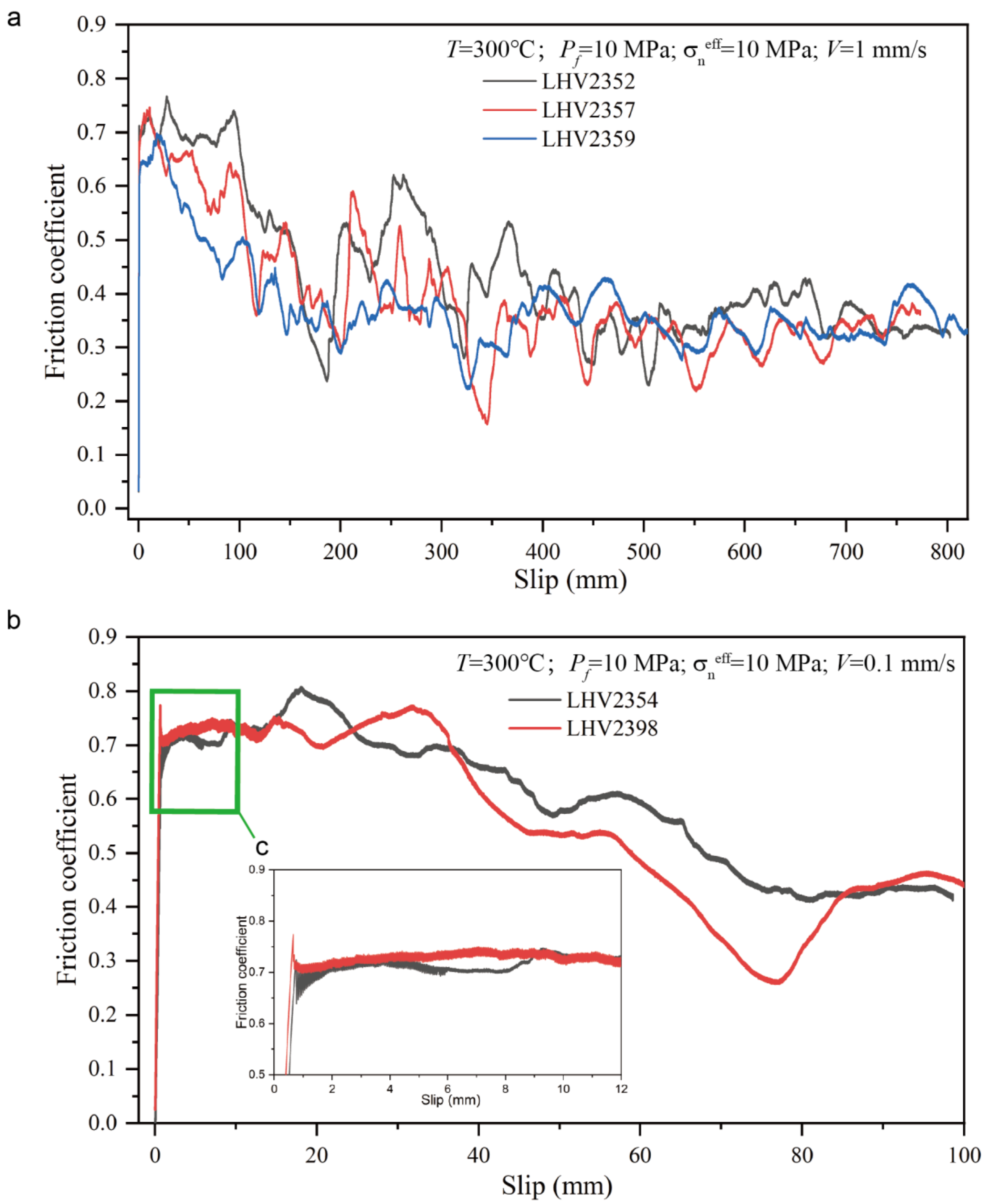

Supplementary Figure 3. Repeated experiments performed under temperature T = 300°C and pore pressure P_f =10 MPa, at slip velocities of 1 mm/s and 0.1 mm/s (water in the liquid state) verifying the reproducibility. a. V = 1 mm/s data. For these experiments, the friction coefficient (μ) has a similar evolution: μ increases to a peak at the beginning of the slip and then gradually decays to a steady-state value of ~ 0.35 over a displacement of 400 mm. **b.** V = 0.1 mm/s data. After the initial elastic loading, μ remains at 0.7-0.75 for ~ 35 mm displacement, then decreases to a lower value of ~ 0.4 after ca. 90 mm of displacement. **c.** Zoom of the initial slip stage.

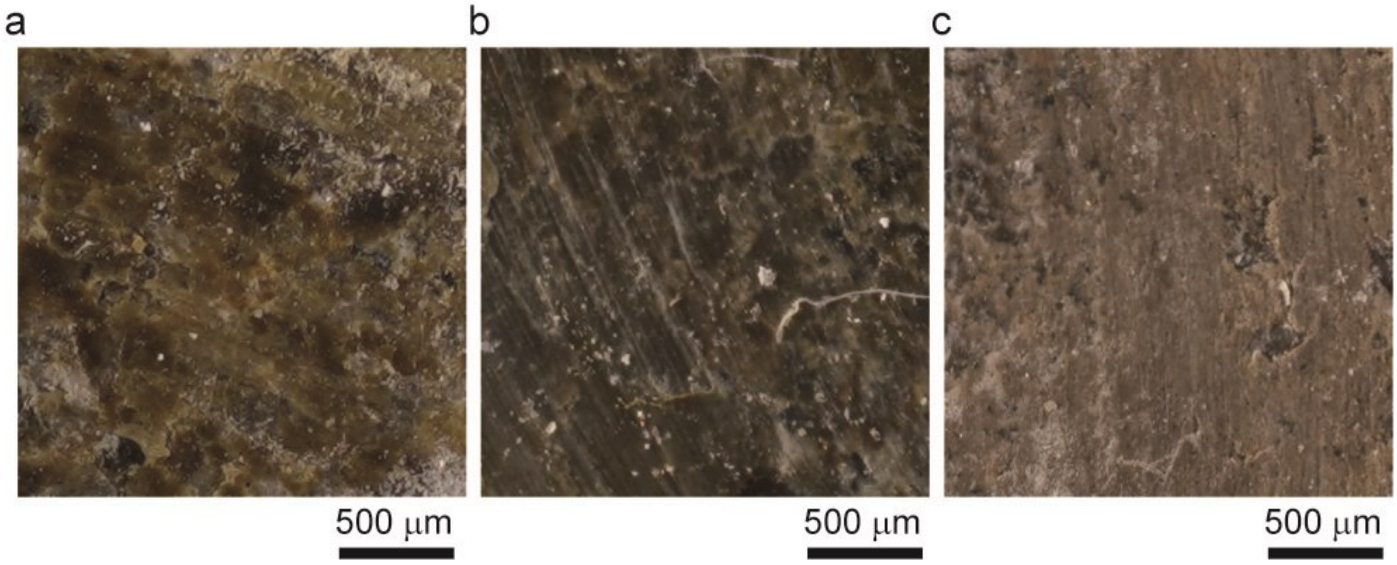

Supplementary Figure 4. "Close up" photographs of slip surfaces recovered from the experiments performed at a slip velocity of 10 μm/s. The slip surfaces are polished and striae are parallel to the slip vector. **a.** Experiment LHV2398, water in liquid state. **b.** Experiment LHV2380, water in supercritical state. **c.** Experiment LHV2391, water in vapor state.

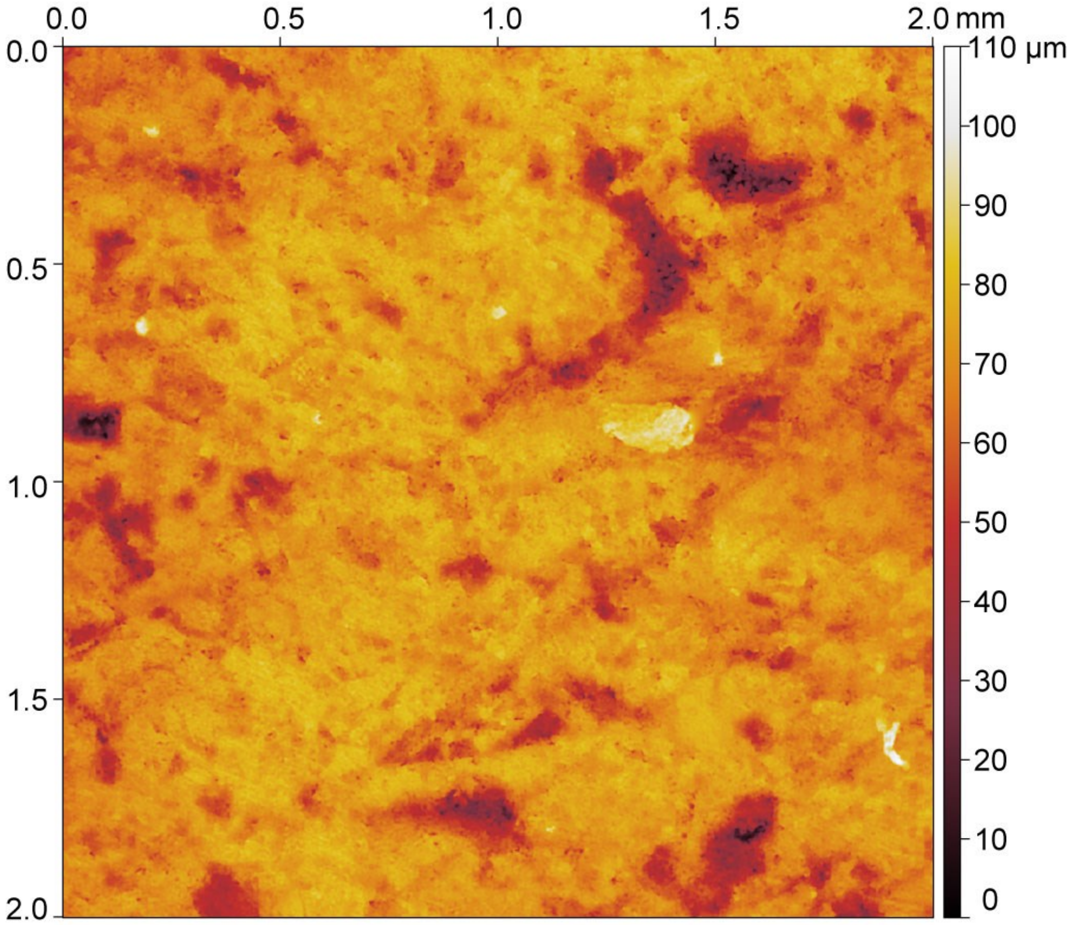

Supplementary Figure 5. Roughness of the starting surface measured by optical profilometer. The root mean square (RMS) value is 5.7 μm.

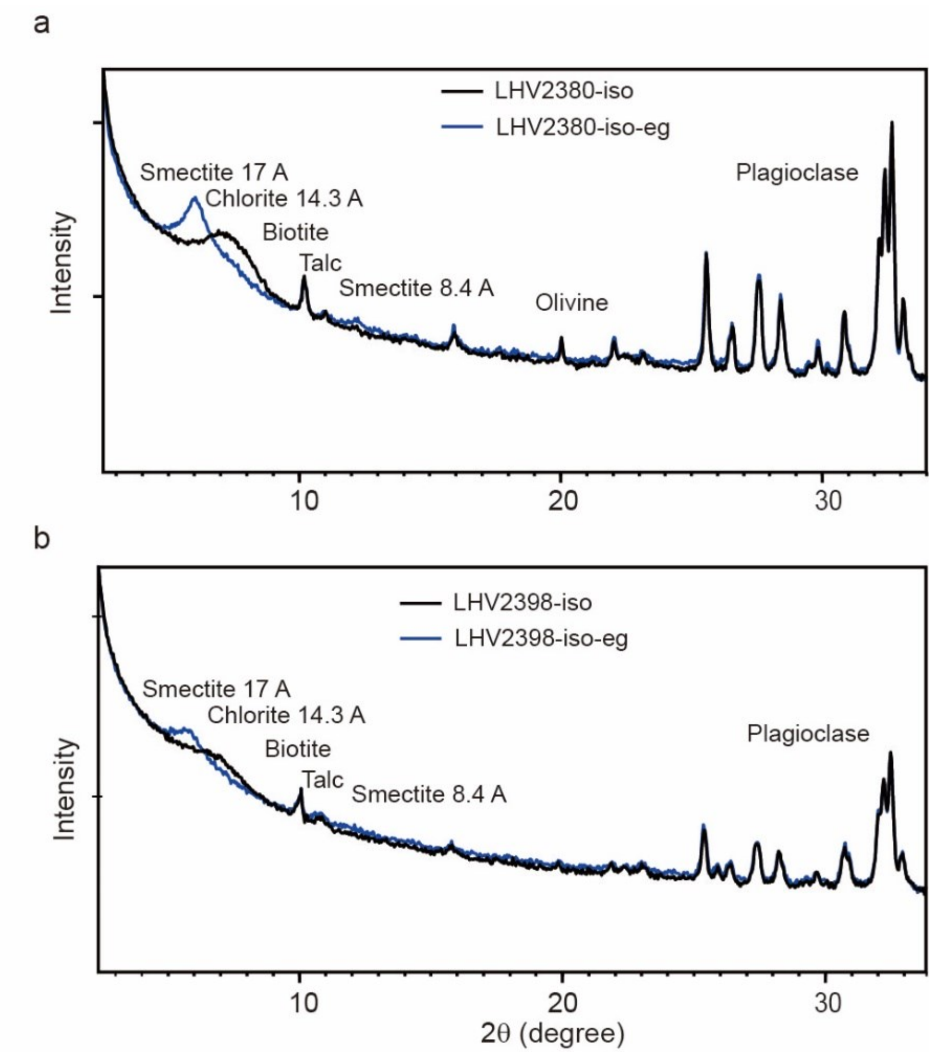

Supplementary Figure 6. XRD spectra after iso-oriented and ethylene glycol treatment of 10 mg of powders recovered from the slip zones of experiments LHV2380 and LHV2398. The spectra show that a mixture of smectite and chlorite is present in both the slip zones. **a.** Experiment LHV2380, water in supercritical state. **b.** Experiment LHV2398, water in liquid state.

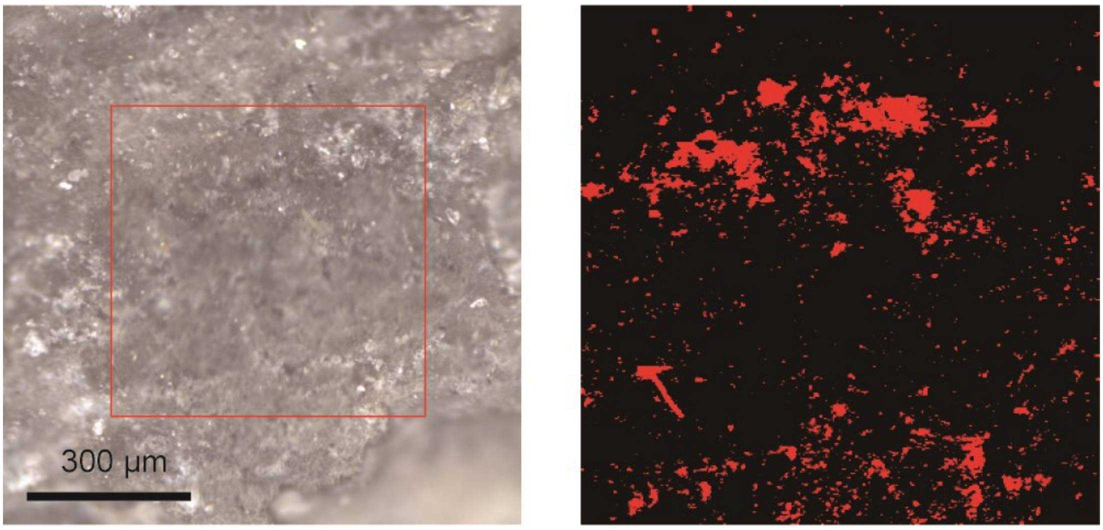

Supplementary Figure 7. Distribution of H-O-H bending in the slip zone for experiment LHV2359 performed with water in the liquid state. Left: the scanned areas of the Raman map are marked with a red in color square. The Raman map (right) show that the H-O-H bending bonds approximatively overlap the striations of the slip surface.

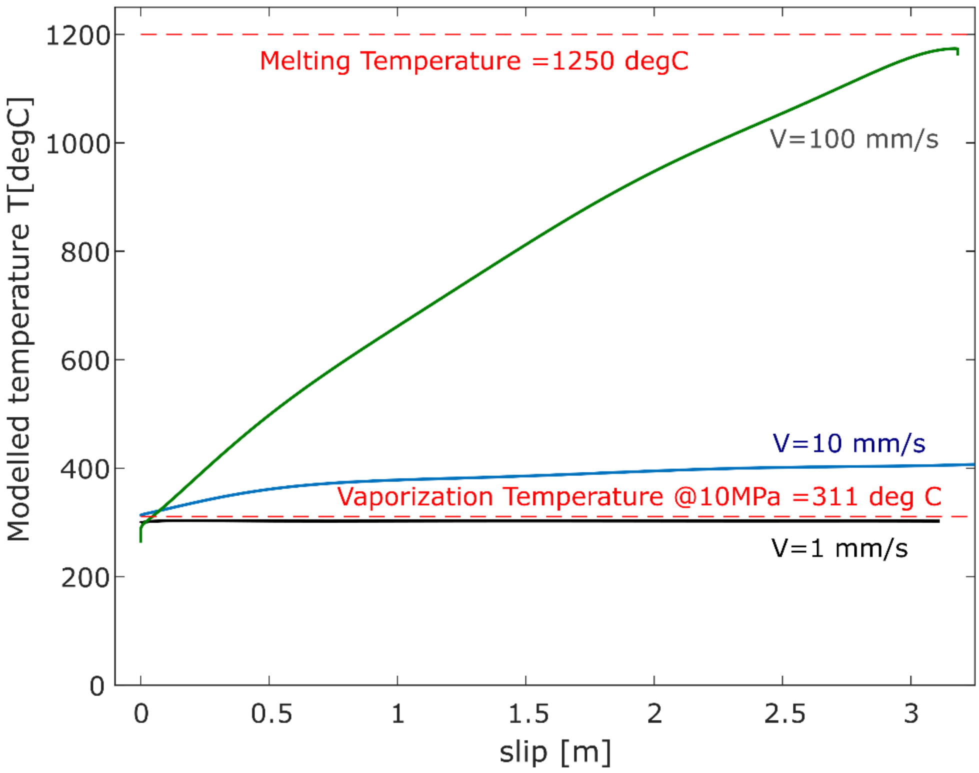

Supplementary Figure 8. Numerical model results of the evolution of temperature in the slipping zone with slip displacement. The liquid-vapor transition temperature of water at pore pressure P_f of 10 MPa is 311°C and is indicated by red in color dashed line. The modelled maximum temperatures for the experiment (LHV2359) preformed at slip velocity V = 1 mm/s, for the experiment (LHV2358) at V = 10 mm/s, and for the experiment (LHV2361) at V = 100 mm/s reach ~306, 404 and 1142°C, respectively.

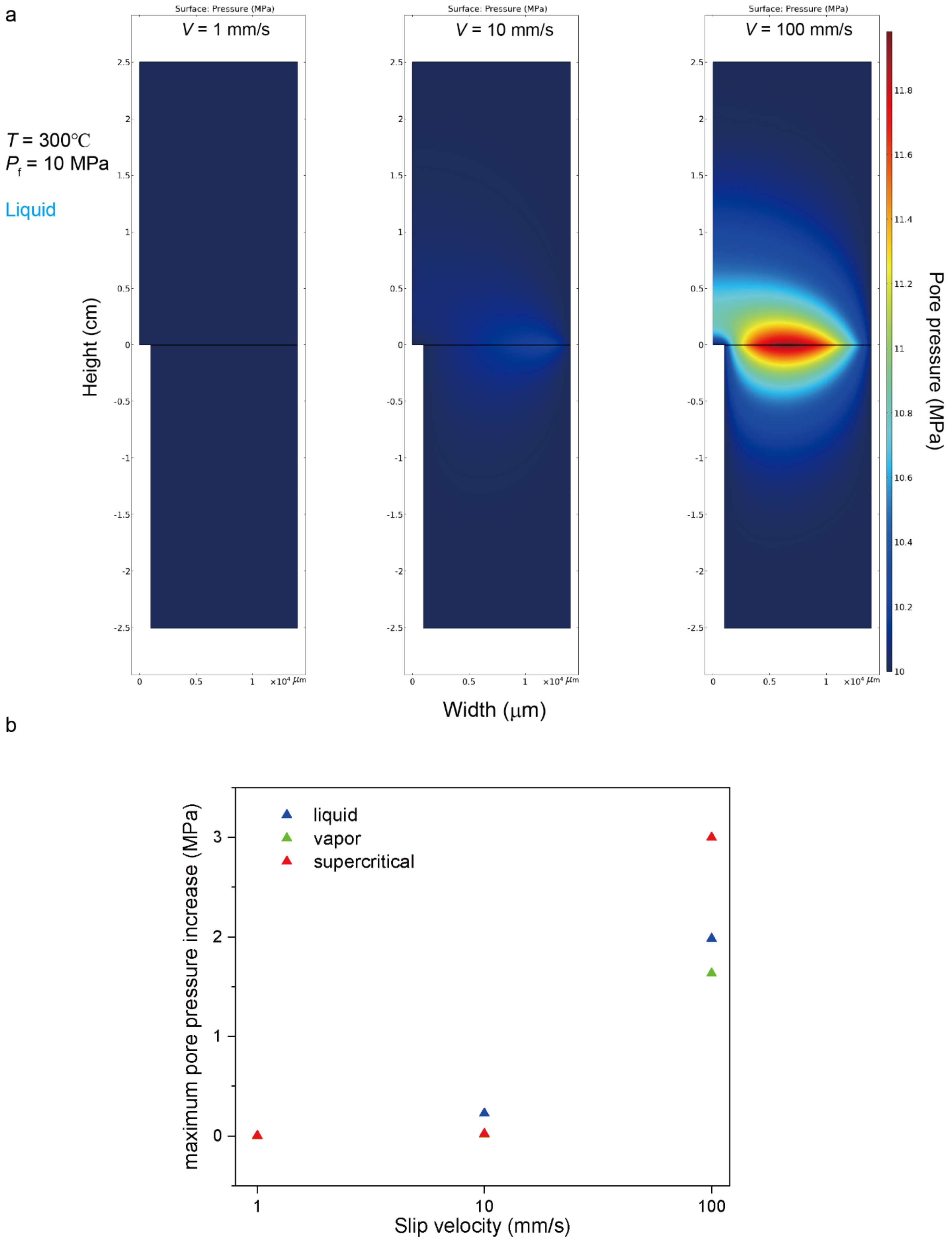

Supplementary Figure 9. Numerical modeling of thermal pressurization (TP). We built a finite element 2-dimentional numerical model with Comsol Multiphysics (For the detailed description of this thermal pressurization modeling[2] see Yao et

al., 2023). The mesh was axisymmetric and included a 50 μm thick homogenous and isotropic slipping zone (see microstructures in Fig. 2 of the main text) with permeability $k = 10^{-18}$ m^2 and porosity $\Phi = 0.1$ (the selection of permeability and porosity roughly follows that reported in Oohashi, et al.[3]). The slipping zone is sandwiched between a pair of low-permeability host rocks. According to the results of the numerical modeling, TP becomes efficient at slip velocity ≥ 100 mm/s, potentially contributing to the observed weakening. **a.** Representative modeling results for experiments performed at 300°C and 10 MPa pore pressure, water in liquid state. **b.** Maximum pore pressure increase vs. slip velocity under hydrothermal conditions.

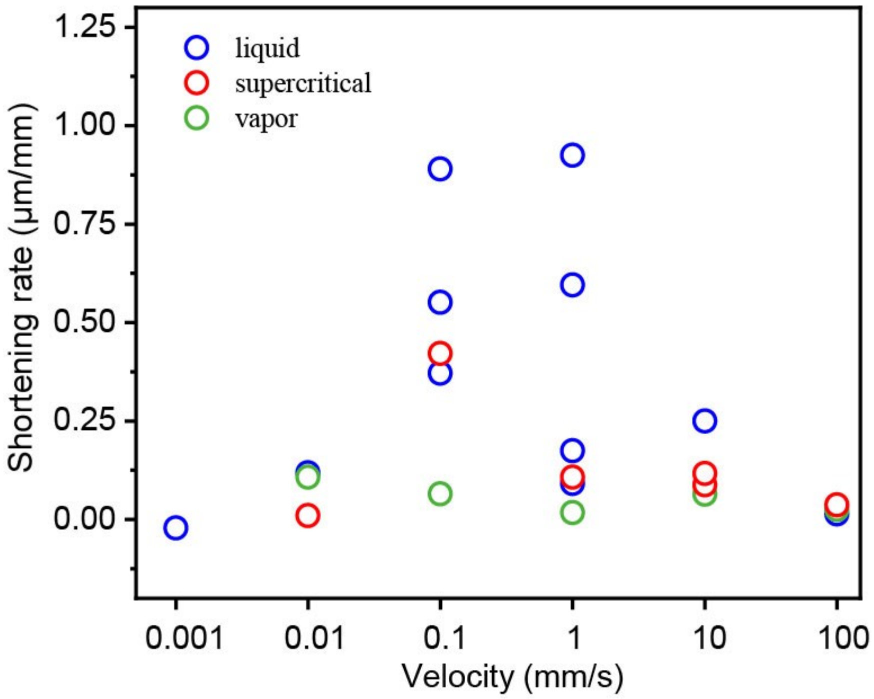

Supplementary Figure 10. Sample shortening rate vs. slip velocity under hydrothermal conditions. In experiments with gabbro samples, at the same effective stresses and regardless of the imposed slip velocity, the measured wear rates are lower in the presence of water in the vapor state than in the presence of water in the liquid and supercritical state.

Supplementary Table 1. XRD Composition of gabbro

Gabbro mineral phases	Phase Quantity (wt.%)	ESD (wt.%)
Biotite	4.0	0.2
Diopside	27.8	0.2
Dolomite	1.5	0.1
Forsterite	12.2	0.2
Lizardite	1.3	0.1
Magnetite	2.6	0.1
Andesine	47.4	0.3
Sanidine	2.8	0.1
Quartz	0.4	0.1

Supplementary Table 2. List of experiments (Summary of experimental conditions and results)

Experiment (LHV)	T (℃)	σ_n^{eff} (MPa)	P_f (MPa)	Phase of water	Velocity (mm/s)	δ (mm)	δ window (mm)	μ_s	μ_p	μ_{ss}	shortening rate (mm/mm)	ΔT (℃)
2379	25	10	10	L	1	3000	2000-2200	0.599	0.721	0.674±0.034	0.033	
2374	300	10	10	L	0.001	380	240-300	0.864	0.864	0.511±0.039	/	
2354	300	10	10	L	0.01	100	80-200	0.742	0.824	0.449±0.012	/	
2398	300	10	10	L	0.01	300	240-300	0.772	0.772	0.261±0.076	0.118	0.04
2349	300	10	10	L	0.1	180	80-180	0.61	0.622	0.370±0.018	0.551	
2356	300	10	10	L	0.1	670	80-200	0.65	0.683	0.442±0.060	0.89	0.42
2400	300	10	10	L	0.1	1000	600-800	0.715	0.797	0.280±0.014	0.371	0.3
2352	300	10	10	L	1	800	600-800	0.71	0.766	0.362±0.029	0.925	
2357	300	10	10	L	1	772	600-772	0.73	0.745	0.328±0.022	0.595	
2359	300	10	10	L	1	3100	2000-2200	0.642	0.695	0.319±0.036	0.174	6.2
2358	300	10	10	L	10	3900	2000-2200	0.621	0.671	0.414±0.016	0.25	104
2360	300	10	10	L	100	3200	2000-2200	0.572	0.572	0.268±0.012	0.014	
2361	300	10	10	L	100	3200	2000-2200	0.574	0.58	0.263±0.027	0.091	842
2391	400	10	10	V	0.01	300	80-200	0.69	0.793	0.695±0.026	0.107	0.07
2388	400	10	10	V	0.1	1000	600-800	0.87	0.915	0.718±0.031	0.065	0.8
2362	400	10	10	V	1	3000	2000-2200	0.793	0.839	0.717±0.010	0.017	6.02
2395	400	10	10	V	10	3000	2000-2200	0.51	0.66	0.469±0.024	0.064	54.4
2363	400	10	10	V	100	3300	2000-2200	0.577	0.637	0.262±0.010	0.025	805.9
2365	400	10	30	SC	0.01	150	80-150	0.381	0.529	0.400±0.013	/	
2380	400	10	30	SC	0.01	300	80-200	0.514	0.558	0.509±0.027	0.01	0.06
2390	400	10	30	SC	0.1	1000	600-800	0.563	0.588	0.281±0.020	0.421	0.31
2367	400	10	30	SC	1	3000	2000-2200	0.531	0.717	0.279±0.038	0.107	2.1
2373	400	10	30	SC	10	3500	2000-2200	0.499	0.658	0.260±0.050	0.088	35.6
2383	400	10	30	SC	10	3000	2000-2200	0.565	0.565	0.296±0.022	0.117	16
2369	400	10	30	SC	100	3000	2000-2200	0.575	0.575	0.212±0.018	0.037	789.2

T: temperature, σ_n^{eff}: effective normal stress, P_f: pore pressure, L: liquid, V: Vapor, SC: Supercritical, δ: slip distance μ_s: static friction, μ_p: peak friction, μ_{ss}: steady-state friction, error bar represents the standard deviation of the data, Distance (δ) window was chosen to calculate the μ_{ss} and shortening rate, ΔT: estimated increase of temperature in the slipping zone.

Supplementary Table 3. Fluid and rock properties. *K* thermal conductivity, *ρ* density, *C* specific heat. Properties were taken from NIST database for thermophysical properties of fluids (based on the IAPWS97 industrial thermodynamic formulation) at different pressures. Gabbro properties from Rempel and Weaver[4].

	K [W/mK]	*ρ* [kg/m³]	*C* [J/(kgK)]
Liquid (T = 577K, P_f = 10 MPa)	0.54841	700	5830
Vapor (T = 675K, P_f = 10 MPa)	0.06878	40	3075
Supercritical (T = 675K, P_f = 30 MPa)	0.32453	250	27000
Gabbro	2.5	2590	1480

References

1 Ma, S., Shimamoto, T., Yao, L., Togo, T. & Kitajima, H. A rotary-shear low to high-velocity friction apparatus in Beijing to study rock friction at plate to seismic slip rates. *Earthq. Sci.* **27**, 469-497, doi:10.1007/s11589-014-0097-5 (2014).

2 Yao, L., Ma, S. & Di Toro, G. Coseismic fault sealing and fluid pressurization during earthquakes. *Nat. Commun.* **14**, 1136, doi:10.1038/s41467-023-36839-9 (2023).

3 Oohashi, K., Hirose, T., Takahashi, M. & Tanikawa, W. Dynamic weakening of smectite-bearing faults at intermediate velocities: Implications for subduction zone earthquakes. *J. Geophys. Res.* **120**, 1572–1586, doi.org/10.1002/2015JB011881 (2015).

4 Rempel, A. W. & Weaver, S. L. A model for flash weakening by asperity melting during high-speed earthquake slip. *J. Geophys. Res. Solid Earth* **113**, doi.org/10.1029/2008JB005649 (2008).
